# Coping Strategies as a Mediator of Posttraumatic Growth among Adult Survivors of the Wenchuan Earthquake

**DOI:** 10.1371/journal.pone.0084164

**Published:** 2013-12-27

**Authors:** Lili He, Jiuping Xu, Zhibin Wu

**Affiliations:** Uncertainty Decision-making Laboratory, Sichuan University, Chengdu, China; Univ of Toledo, United States of America

## Abstract

**Objective:**

By testing the mediating effect of coping strategies on the relationship between social support (SS) and posttraumatic growth (PTG), the aim of this research was to develop a new approach for the study of post-disaster psychological intervention.

**Methods:**

A mediating effect model analysis was conducted on 2080 adult survivors selected from 19 of the counties hardest-hit by the 2008 Wenchuan earthquake. The Social Support Rating Scale and the Coping Scale were used to predict the PTG.

**Results:**

A bivariate correlation analysis showed that there was a correlation between posttraumatic growth, social support and coping strategies. The mediation analysis revealed that coping strategies played a mediating role between social support and posttraumatic growth in survivors after the earthquake.

**Conclusion:**

The results demonstrated that mental health programs for survivors need to focus on the establishment of a good social support network, which was found to be conductive to maintaining and increasing mental health levels. At the same time, adequate social support is able to assist survivors in adopting mature coping strategies, such as problem solving and asking for help. Hence, social support was found to play a vital role in balancing and protecting mental health.

## Introduction

On May 12th 2008, at 14∶28 pm, an earthquake measuring 8.0 on the Richter scale struck Wenchuan, Sichuan Province, China, resulting in enduring physical and mental problems for survivors, many of whom had been left homeless or had experienced the death of a loved one. Natural disasters often result in negative consequences; however, sometimes positive changes, such as posttraumatic growth (PTG) –“a positive change experienced as a result of the struggle with trauma” [1], [2], can arise from such tragedies. PTG can be defined further as a positive psychological development arising out of extremely adverse circumstances. Therefore, exploring how PTG contributes to the experience of recovery and how it affects the efficacy of more mechanistic interventions is an important addition to disaster recovery literature.

Research into disasters has tended to focus on the subsequent negative psychological and behavioral changes, and the related stress reactions [3]–[5]. Recent research into positive outcomes after trauma has heightened attention on this issue, as has emphasized the transformative potential of a person after highly stressful events or circumstances [6]. PTG has been observed in survivors of a wide range of traumatic events, such as the recently bereaved, those suffering from serious medical illnesses, and those who have been exposed to rape, warfare, natural disasters and [7]–[10] and military combat [11]. However, most PTG literature has centered on the survivors of serious medical illnesses, with very few examining earthquake survivors [12], [13]. PTG literature on earthquake survivors has hypothesized that PTG plays a similar role in terms of psychological intervention to that in survivors of serious medical illness.

Posttraumatic growth is defined as the subjective experience of a positive psychological change reported by an individual as result of a struggle with trauma. The functional-descriptive models of PTG discussed in [1], [8] give examples of survivors who have an increased appreciation of life, the ability to set new life priorities, a sense of increased personal strength, the ability to identify new possibilities, an improved closeness in intimate relationships, or a positive spiritual change [14].

The life crises and personal growth models [15]–[16] presented in this research revealed the importance of coping strategies and social support, as environmental and personal factors can influence cognitive appraisal processes and coping responses which, in turn, can affect the outcome of the crisis. A two-component-model [17] demonstrated that PTG had a constructive side (e.g. self-transcending) and also a dysfunctional side (e.g. illusory, self-deception), and explained that at different times different coping strategies are adopted.

Researchers have attempted to capture a measure of PTG using qualitative and quantitative methodologies. Consequently, seven instruments have been developed to measure this growth. The most widely used [18] is the Posttraumatic Growth Inventory [7] as it is standardized, and has the highest reliability and validity [19] and shows good applicability in both basic and clinical research.

A significant amount of research has found that coping strategies and PTG are related [7], [20]. Studies have also found that coping strategies appear to have a direct impact on PTG, and play a mediating role between the perception of social support and PTG [21]. Social support also seems to positively influence PTG [20].

Psychologists often conduct research to establish whether and to what extent one variable affects another. However, the discovery that two variables are related to each other is only a small part of the psychology’s aim. Of even greater focus for researchers is explaining how or by what means a causal effect occurs, so a variable which may be called a mediator “to the extent that it accounts for the relation between the predictor and the criterion” [22] has become an important focus for researchers. There is a large and growing body of research on methods for the testing of simple mediation hypotheses and up till now at least a dozen methods have been proposed [23]. In the last two decades, Baron and Kenny's causal steps approach [22] and the Sobel test [24] have been regularly applied in the biomedical and social sciences for mediation analysis. Even so, these approaches have several limitations [25] and bootstrapping techniques are now being recommended to test mediation [26], [27], [28]. Prior simulation studies demonstrated that bootstrapping is a more powerful and accurate method than causal steps and the Sobel test in the estimation of the mediation effect [29]. In this paper, Baron and Kenny's causal steps approach and bootstrapping techniques are combined to test the mediation effect [30].

To the best of our knowledge, there has been little research which focuses on the relationship between social support, coping strategies and PTG after an earthquake. However, as mentioned, PTG has been shown to be a vital factor in psychological intervention. The aim of our research is to test and explore the relationships between PTG, social support and coping strategies in Wenchuan earthquake survivors. Within the framework of the Schaefer and Moos (1998) model, a model is developed and the variables predicting PTG in Wenchuan earthquake survivors tested. The model has two variables (social support and coping strategies) which are treated as independent variables, and PTG which is treated as a dependent variable. Specifically, we hypothesize that:

1. The three domains of Social support and PTG have a significant correlation.
2. Coping strategies have a major effect on mediating the association between the social support and PTG of earthquake survivors.

## Methods

### Ethics Statement

The data were analyzed anonymously. The study and procedures were approved by the ethics committee of Sichuan University and written informed consent was obtained from each subject after a full explanation of the study procedures. The investigation was conducted in accordance with the latest version of the Helsinki Declaration.

### Procedure

Data collection for the present study was conducted one year after the Sichuan earthquake. In order to ensure that the survey was systematically developed, we established a research team composed of a psychology expert, professional English translators, and doctoral and masters students. Effective and feasible questionnaires were designed and a pilot test carried out in May and June, 2009, with a pool of 118 workers participating. Minor modifications and adjustments were made according to the pilot test feedback. A final version of the questionnaire was used in the formal investigation.

In order to ensure that the sample would be significantly representative and to reduce statistical error, we adopted a stratified sampling methodology to collect the relevant data. In the first sampling stage, the following 19 hardest-hit counties of this earthquake were selected due to their high degree of exposure to this earthquake; Dujiangyan, Pengzhou, Chongzhou, Shifang, Mianzhu, Jiangyou, Anxian, Pingwu, Beichuan, Jiange, Qingchuan, Hanyuan, Wenchuan, Lixian, Maoxian, Songpan, Heishui, Xiaojin and Lueyang. In the second stage, to ensure that the sample was representative, we applied simple random sampling to each layer. The sample size, therefore, depended on the number in each layer in relation to the respective population size.

The research team was divided into 19 smaller groups, with each one being responsible for one county. Each group was made up of 2 graduate students, an outstanding volunteer and a staff member from the local government. The team members were then trained over 5 days. The training content included related psychological knowledge, and an explanation as to how to investigate and deal with respondents’ problems. Very few respondents had either a low education level or literacy problems. For those with literacy or understanding problems, volunteers used oral and written methods to assist them in completing the survey. All participants were assured of their confidentiality and all took part voluntarily.

### Sample

The sample for the present study was drawn mainly from workers in varying occupations, such as engineering, medicine, teaching, and agriculture. We asked the relevant government departments for help, which ensured a high response rate. A total of 2300 individuals were involved in this survey and 2080 completed the questionnaire, a response rate of 90.4%.

The study sample demographic characteristics are given in Table 1. The mean age of the 2,080 participants at the time of the interview was 38.24±8.82 years (ranging from 18 to 68 years).

**Table 1:** The characteristics of the study sample.

	N	%		N	%
Gender			Education level		
Male	1227	59	High school or below	1095	52.6
Female	853	41	College graduate	938	45.1
Age groups			Post-graduate	47	2.3
18–24	74	3.6	Income (RMB/month)		
25–34	584	28.1	<1000	385	18.5
35–44	852	41	1000–2000	1360	65.4
45–54	491	23.6	>2000	335	16.1
55–68	79	3.8			
Ethnicity group					
Han	1674	80.5			
Tibetan	147	7.1			
Qiang	211	10.1			
Hui	37	1.8			
Other	11	0.5			

### Instruments

The self-report questionnaire covered items on demographic characteristics (including gender, age, ethnicity, education level and income), posttraumatic growth, social support and coping strategies. All assessment forms were translated from English to Chinese and back-translated by a bilingual team of professionals.

### PTG

The assessment of a positive post disaster outcome was evaluated using the Posttraumatic Growth Inventory (PTGI) [7]. This inventory consists of 21 items across five major domains: relating to others (7 items), new possibilities (5 items), personal strength (4 items), spiritual changes (2 items), and appreciation of life (3 items). Cultural differences can also affect posttraumatic growth. Therefore, when using the PTGI a number of items and structural domains needed adjustment to be accordance with the reality on the ground. Further research showed that the religious beliefs of participants had little effect on PTG, and a devastating trauma raised doubts about the omniscient object of their belief, so two items (“A better understanding of spiritual matters” and “I have a stronger religious faith”) were excluded in this study. Questionnaires were scored using a scale from “no change” to “a complete change” across a 5 point scale (0 for no change and 5 as complete change). Higher scores represented higher levels of posttraumatic growth. The total PTGI score was the sum of the 19 items, with a total score of 95. For this study, the internal reliability of this instrument was 0.88. The internal reliabilities of the four subscales were 0.83, 0.79, 0.81, and 0.80 respectively [13].

### Social Support

Social support has been defined as the ‘assistance and protection given to others, especially to individuals’. In our study, 10 questions were designed according to Xiao’s 1999 work, which were adjusted for the Wenchuan earthquake. The item scores for the Social Support Rating Scale (SSRS) were simply added up, generating a total support score ranging from 12 to 66, a subjective support score ranging from 8 to 32 (4 questions), an objective support score ranging from 1 to 22 (3 questions), and a support availability score ranging from 3 to 12 (3 questions), respectively. Higher scores indicated stronger social support. The Cronbach alpha coefficient for social support was 0.91 [31]

### Coping Strategies

The coping strategies were evaluated using the Coping Scale (CS) developed by Xiao and Xu, which is a widely applicable self-report measure for situational coping and encompasses problem avoidance, problem solving and social support seeking strategies [32]. The CS consists of six domains; problem solving, self-blame, asking for help, having fantasies, problem avoidance and rationalization. There are 36 items, and each item is answered using a 5-point Likert scale where 1 = not sure at all and 5 = very sure. The questionnaire has good reliability and validity. In this study, the CS grand score was produced using a Cronbach’s alpha of 0.879 and a Split-Half of 0.865 in a reliability test [33].

### Statistical Analysis

In the present study, descriptive statistics, variance analysis, and correlation analysis were selected. Multiple regression analysis was the principal data analysis technique used to examine the hypotheses. For missing data, this study adopted a linear trend at point and unbiased estimation. All tests were 2-tailed, and significance was set at 0.05. All statistical procedures were completed using SPSS 16.0 (SPSS, Chicago, IL, USA).

**Figure 1:**
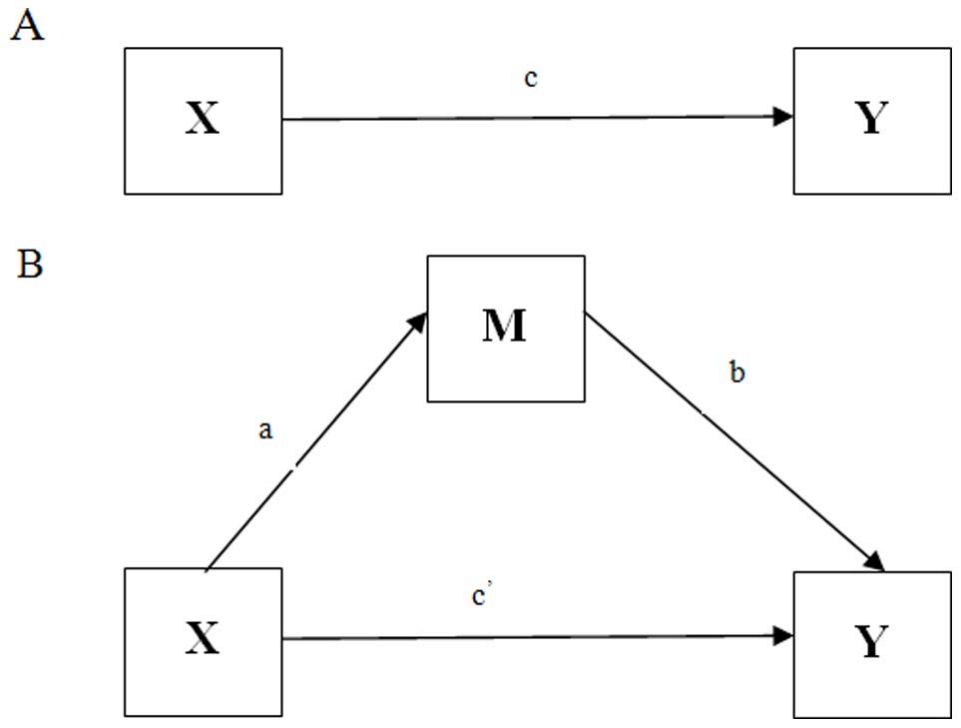
A: Illustration of a direct effect. X affects Y. B: Illustration of a mediation design. X affects Y indirectly through M.

Mediation hypotheses posit how, or by what means, an independent variable (X) affects a dependent variable (Y) through one or more potential intervening variables or mediators (M) (See Figure 1). Bootstrapping, a nonparametric resampling procedure which does not impose an assumption of normality on the sampling distribution, is an additional method for testing mediation. Bootstrapping is a computationally intensive method that involves repeatedly sampling from the data set and estimating the indirect effect in each resampled data set. Using the mean of these N time estimates, the indirect effect was computed. If the 95% indirect affect bias corrected and accelerated confidence intervals (95% CI) did not contain zero, then the indirect effect was considered statistically significant [34].

## Results

### Survey Responses

Grouped by demographic variables, the PTG scores are shown in Table 2. Only the gender group showed different PTG evaluations. Females reported higher PTG scores than males. Subjects of different age groups also reported different PTG scores. In terms of ethnicity, the Qiang ethnic group had the highest score. As education levels increased, PTG scores showed a positively increasing trend. Subjects from different income groups also reported different PTG scores. There were no statistically significant associations between PTG and ethnicity and income (P>0.05). Recall that in all subgroups, a higher score indicated a better PTG level.

**Table 2:** Scores of PTG-Total by background characteristics.

	Mean (SD)	p value		Mean (SD)	p value
Gender		0.01	Education level		0.07
Male	57.85 (7.27)		High school or below	58.16 (7.25)	
Female	58.84 (7.50)		College graduate	58.49 (7.54)	
Age		0.003	Post-graduate	58.54 (6.43)	
18–24	57.92(6.24)		Income (RMB/month)		0.016
25–34	58.18 (7.86)		<1000	58.52 (7.41)	
35–44	58.84 (7.27)		1000–2000	58.42 (7.62)	
45–54	57.56 (7.06)		>2000	57.11 (6.06)	
55–65	56.72 (7.14)				
Ethnicity group		0.34			
Han	58.14 (7.41)				
Tibetan	58.69 (7.04)				
Qiang	58.95 (7.51)				
Hui	57.86 (5.59)				
Other	56.82 (7.84)				

Note. PTG = posttraumatic growth.

### Correlations between Study Variables

Bivariate correlation analysis was used to analyze the relationship between the study variables presented in Table 3. There was a moderate correlation between PTG and the three social support domains. The results clearly support the hypothesis that the three social support domains and PTG have a significant correlation. The three social support domains were also found to correlate with each other. There was a moderate correlation between PTG and having fantasies (HF) (r = −0.390), self-blame (SB) (r = 0.29), asking for help (AH) (r = 0.22), and rationalization (RA) (r = −0.15). There was a high correlation between PTG and problem avoidance (PA) (r = 0.58) and problem solving (PS) (r = 0.52). The six coping strategies domains correlated significantly with each other.

**Table 3:** Correlation coefficients among the study variables.

	1	2	3	4	5	6	7	8	9	10
**PTG**	1									
**SS**	.17**	1								
**OS**	.21**	.51**	1							
**SA**	.30**	.24**	.55**	1						
**PA**	.58**	.17**	.13**	.03*	1					
**HF**	−.39**	.18**	.14**	.03*	.40**	1				
**SB**	.29**	.16**	.16**	.04*	.23**	−.24**	1			
**AH**	.22**	.22**	.18**	.08**	.28**	−.33**	.25**	1		
**RA**	−.15**	.18**	.09**	.05*	.22**	−.26**	.26**	.29**	1	
**PS**	.52**	.18**	.18**	.04*	.32**	−.32**	.56**	.37**	−.24**	1

Note. PTG = post-traumatic growth; SS = subjective support; OS = objective support, SA = support availability; PA = problem avoidance; HF = having fantasies; SB = self-blame; AH = asking for help; RA = rationalization; PS = problem solving;

P<0.05;

P<0.01.

### Testing the Coping Strategies Mediation Effect Model

In this study, the independent variable was social support (_X_), the mediating variable was the coping strategies (M), and the dependent variable was PTG (_Y_). All variables were centered, so the mediation relationship used regression equations as shown below:
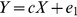
(1)

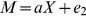
(2)

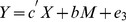
(3)


A hierarchical regression analysis was conducted to determine the significant PTG associations (See Table 4). The findings revealed that social support and PTG were significantly and positively associated, and social support (Step 1) predicted PTG, whereby as social support increased by one unit, the PTG increased by 0.135 unit. The regression model accounted for 18% of variance in the PTG (F>F_0.05_, p<0.05, p<0.05, BCa95% CI: 0.100, 0.220).

**Table 4:** The results of hierarchical regression analysis.

	F	R^2^	SE	coefficient	BCa 95% CI	
					Lower	Upper
Step1						
Social support	38.422***	0.18	0.030	0.135	0.100	0.220
Step2	42.627***	0.39				
Social support			0.030	0.107	0.062	0.191
Coping strategies			0.087	0.148	−0.735	−0.372

Note. 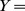
post-traumatic growth (PTG); 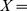
social support; 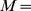
coping strategies;

P<0.05;

P<0.01;

P<0.001.

**Figure 2:**
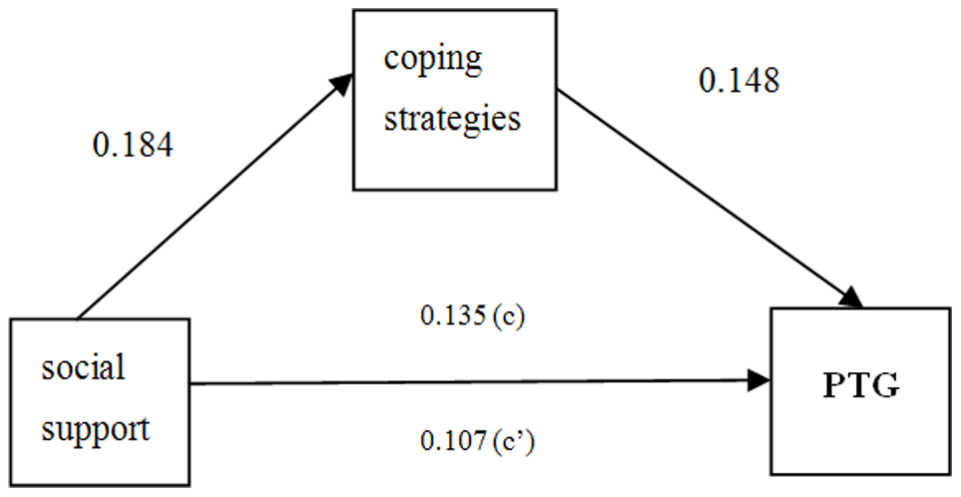
Results of the proposed mediation model. Predictor: 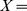
social support, Mediator: 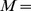
coping strategies, Outcome variable: 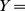
post-traumatic growth (PTG). Coefficients are unstandardized parameter estimates.

The direct effect of social support on an increase in PTG also remained significant once coping strategies were added to the model (Step 2) (F >F_0.05_, p<0.05,BCa95% CI: −0.735, −0.372, See Table 4), indicating that an increase in coping strategies mediated, in part, the influence of social support on the PTG increase. Overall, independent variables explained 39% of the dependent’s variance. An examination of the indirect effect (ab path) revealed significant mediation (BCa95% CI: −0.072, −0.045) [30]. A Sobel test showed that the inclusion of coping strategies were significantly influenced by social support (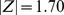
>0.90, p<0.05) [24], [35], thus coping strategies were tested as a mediator of the association between social support and PTG (See Figure 2).

To estimate the mediation pathway effect size, we calculated the proportion of the total effect of the independent variable (social support) on the dependent variable (PTG) that was mediated by the coping strategies with the formula 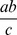 [36]. The coping strategies meditation proportion was 25.45%.

## Discussion

In line with previous studies [7], this study demonstrated that females report more growth than males. Previous research revealed that different ages appear to have different relationships with PTG [37]–[38], but this study found that, as people got older, the individual posttraumatic growth experience increased at first and then gradually declined. One possible reason for this might be that up to 44 years old, people are more likely to accept challenges and use this as a personal growth opportunity, but after 44 people may become less willing to accept challenges and take risks. Previous PTG research found some evidence of an ethnicity-PTG association [39]. In this study it was found that the Qiang Ethnic group had the highest PTG of all minority populations. The distinction between this research and previous research in the PTG across ethnic groups was attributed to the diverse cultural beliefs leading to different interpretations of the growth experience.

Some previous research found that those with less education were correlated with higher growth [37], [40]. Other research has reported that education level and PTG have no significant relationship [38]. This study has demonstrated that the higher the education level, the higher the PTG level. This research objective was limited to earthquake trauma, so the education level of the survivors may assist in trauma understanding, which could improve confidence in physical and mental health recovery. Previous research also indicated that economic status was related to PTG [37], [41]. This study found that the lower the income, the higher the PTG level. The reason for this may be that those survivors on a lower income are more likely to have a higher premium on the perception of social support, so consequently, reported a higher PTG.

In line with previous research [7], [17], [18], [21], [42], social support had a positive influence on PTG. This study revealed that social support was positively related to coping strategies, which is consistent with previous research [16], [43], [44]. A supportive environment encourages positive beliefs and assists survivors in the adoption of positive coping strategies. From this, it can be surmised, therefore, that a higher level of social support leads to a higher level of physical and mental health.

Some studies have found that positive coping strategies and PTG were related [7], [19], [20], [45], [46] while other studies have negated this [39]. In our study, we examined the effect of social support on PTG instead of the perceived social support suggested, and we adopted six different coping strategies domains instead of three. From these adjustments, we found that PTG was positively related with AH, PS, PA and SB, and negatively correlated to HF, RA, but the difference was not significant. The reason that this study has come to a different conclusion is that problem avoidance has both a positive and negative impact on the process of successfully coping with trauma. Positively, PA was necessary for an individual to recover from the trauma, which accorded with after trauma psychological development and SB had a positive and negative effect. When faced with natural disasters, a positive SB was a type of inner relief or self-soothing, so it played a positive role in PTG.

Some studies have indicated that there were indirect relationships between perceived social support and coping strategies [21]. Coping is accepted as “a transactional process between individuals, the context, and post-trauma outcome” [47]. In this study, this indirect relationship was examined by evaluating how coping strategies played a mediating role between social support and PTG. The results revealed that survivors maybe even more likely to report higher PTG once they gain more social support. That is, Chinese people believed they were bound to have access to social support from the outside after the earthquake, which means that the survivors were more likely to report PTG and to employ appropriate coping strategies.

## Conclusion

Even though individuals have been shown to deviate from reality under certain circumstances, their PTG is still adaptable. This belief in growth can possibly assist in offsetting the negative consequences of trauma. Therefore, psychological intervention which promotes PTG should be adopted after disasters. This study analyzed the mediating effect of coping strategies on the relationship between social support and PTG, and proposed a new approach for mental health intervention.

Firstly, it is necessary to pay attention to the establishment of a good social support network, which can provide sufficient emotional communication and material help from the outside whilst giving encouragement and affirmation, leading to survivors being able to overcome any growth difficulties. Therefore, stress is reduced and a higher PTG level obtained. Thus, the level of harm resulting from the pressure post trauma can be weakened, which is conductive to maintaining and improving mental health. Secondly, significant social support can assist trauma survivors adopt proper coping strategies, such as PA and SB, which can assist in relieving mental stress, and solving problems. Therefore, social support could be seen to play a vital role in balancing and protecting mental health.

The limitations of this study lie in the fact that no comparisons have been made with pre-earthquake data due to the lack of data. Therefore, it is difficult to definitively make conclusions about the extent of the effect of the earthquake. Results from this study could provide a theoretical basis for effective post-disaster psychological intervention, and have certain practical significance in the promotion of mental health programs in disaster areas. Future research should further explore other factors affecting PTG.
